# Dialysis-related constrictive pericarditis: old enemies may sometimes come back

**DOI:** 10.1590/2175-8239-JBN-2020-0252

**Published:** 2021-07-07

**Authors:** Precil Diego Miranda de Menezes Neves, Fábio Cerqueira Lario, Sara Mohrbacher, Bernadete Maria Coelho Ferreira, Victor Augusto Hamamoto Sato, Érico Souza Oliveira, Leonardo Victor Barbosa Pereira, Alessandra Martins Bales, Luciana Loureiro Nardotto, Jéssica Nogueira Ferreira, Lívia Barreira Cavalcante, Pedro Renato Chocair, Américo Lourenço Cuvello-Neto

**Affiliations:** 1 Hospital Alemão Oswaldo Cruz, Centro de Nefrologia e Diálise, São Paulo, SP, Brasil.; 2 Universidade de São Paulo, Faculdade de Medicina, Divisão de Nefrologia, São Paulo, SP, Brasil.; 3 Hospital Alemão Oswaldo Cruz, Centro de Cardiologia, São Paulo, SP, Brasil.; 4 Hospital Alemão Oswaldo Cruz, São Paulo, SP, Brasil.; 5 Universidade de São Paulo, Faculdade de Medicina, Divisão de Patologia, São Paulo, SP, Brasil.

**Keywords:** Cardiovascular Surgical Procedures, Dialysis, Renal Dialysis, Hypotension, Pericarditis, Uremia, Procedimentos Cirúrgicos Cardiovasculares, Diálise, Diálise Renal, Hipotensão, Pericardite, Uremiakeywords

## Abstract

Cardiovascular disease is the main cause of death in patients with chronic kidney disease (CKD). Several heart conditions have been associated with CKD, including myocardial and pericardial diseases. This paper describes a case of Dialysis-related constrictive pericarditis in a patient diagnosed with sudden hypotension during a hemodialysis session. A 65-year-old man diagnosed with hypertension, diabetes, obesity, and cirrhosis on hemodialysis for two years complained of symptoms during one of his sessions described as malaise, lipothymia, and confusion. The patient had a record of poor compliance with the prescribed diet and missed dialysis sessions. He was sluggish during the physical examination, and presented hypophonetic heart sounds, a blood pressure of 50/30mmHg, and a prolonged capillary refill time. The patient was referred to the intensive care unit and was started on antibiotics and vasoactive drugs. His workup did not show signs of infection, while electrocardiography showed low QRS-wave voltage. His echocardiogram showed signs consistent with a thickened pericardium without pericardial effusion. Cardiac catheterization showed equalization of diastolic pressures in all heart chambers indicative of constrictive pericarditis. The patient underwent a pericardiectomy. Examination of surgical specimens indicated he had marked fibrosis and areas of dystrophic calcification without evidence of infection, consistent with Dialysis-related constrictive pericarditis. Hypotension for unknown causes must be considered in the differential diagnosis of dialysis patients.

## RESUMO

A doença cardiovascular é a principal causa de morte em pacientes com doença renal crônica (DRC). Várias formas de acometimento cardíaco têm sido associadas. à DRC, incluindo doenças miocárdicas e pericárdicas. Este artigo descreve um caso de pericardite constritiva relacionada a em um paciente diagnosticado com hipotensão súbita durante uma sessão de hemodiálise. Um homem de 65 anos com diagnósticos prévios de hipertensão, diabetes, obesidade e cirrose em hemodiálise por dois anos queixou-se de sintomas durante uma de suas sessões, descritos como mal-estar, lipotímia e confusão mental. Apresentava histórico de baixa adesão à dieta prescrita e faltas frequentes às sessões de diálise. Ele estava fraco durante o exame físico e apresentava bulhas cardíacas hipofonéticas, pressão arterial de 50/30mmHg e tempo de enchimento capilar prolongado. O paciente foi encaminhado para a unidade de terapia intensiva e iniciou o tratamento com antibióticos e drogas vasoativas. Investigação laboratorial não mostrou sinais de infecção, enquanto o eletrocardiograma mostrou baixa voltagem de complexo QRS. Seu ecocardiograma evidenciou sinais consistentes com um pericárdio espessado, sem derrame pericárdico. O cateterismo cardíaco mostrou equalização das pressões diastólicas em todas as câmaras cardíacas, indicativo de pericardite constritiva. O paciente foi submetido a uma pericardiectomia. O exame anatomopatológico mostrou sinais de acentuada fibrose acentuada fibrose e áreas de calcificação distrófica sem evidência de infecção, consistente com pericardite constritiva relacionada a por diálise. A hipotensão por causas desconhecidas deve ser considerada no diagnóstico diferencial de pacientes em diálise.

## INTRODUCTION

Cardiovascular disease ranks as the top cause of death of patients with chronic kidney disease (CKD) worldwide^1^^-^ 3. Other heart conditions have also been associated with kidney failure, including ventricular hypertrophy, myocardiopathy, valvopathy, arrhythmia, and pericarditis^4^^-^ 6.

Pericardial involvement in patients with CKD is multifactorial and generally related to conditions leading to ineffective dialysis such as hypercatabolic status and low dose of delivered therapy^7^^,^ 8. In this context, chronic exposure to a uremic environment leads to inflammatory alterations in the pericardium and to the subsequent onset of fibrosis and constrictive pericarditis^4^^,^ 9.

This paper reports a case of dialysis-related constrictive pericarditis, and specifically addresses clinical presentations, diagnosis, and treatment.

## CASE PRESENTATION

A 65-year-old man on hemodialysis complained of symptoms during one of his sessions described as malaise, lipothymia, and confusion. He did not report dyspnea, tachycardia, chest pain or other symptoms. He had been previously diagnosed with hypertension, diabetes, morbid obesity, and cirrhosis secondary to non-alcoholic steatohepatitis. The patient was on allopurinol 100 mg/day, sertraline 50 mg/day, esomeprazole 40 mg/day, and sevelamer 2.4 g thrice daily.

The patient had been on hemodialysis for two years for diabetic nephropathy, but frequently missed sessions. He had no residual urine output and a long record of high interdialytic weight gain and poor compliance with his prescribed diet. The patient had a radiocephalic arteriovenous fistula on his right wrist. In order to address his frequent absences, he was prescribed hemodialysis six times a week with 2.5-hour-long sessions. The dialyzer was equipped with a high-flux polysulfone membrane with a surface area of 2.2 m^2^, with Qb: 370 mL/min and Qd: 800 mL/min. Dialysate prescription: Ca: 2.5 mEq/L; K: 1 mEq/L; Na: 138 mEq/L; bicarbonate: 36 mmol/L; unfractionated heparin: 5000 IU/session. If the patient had attended his sessions as prescribed, his calculated standard Kt/V would have been 2.45. However, since he only came to the clinic for three or four sessions a week, his actual KT/V was 1.5-2.

**Figure 1:**
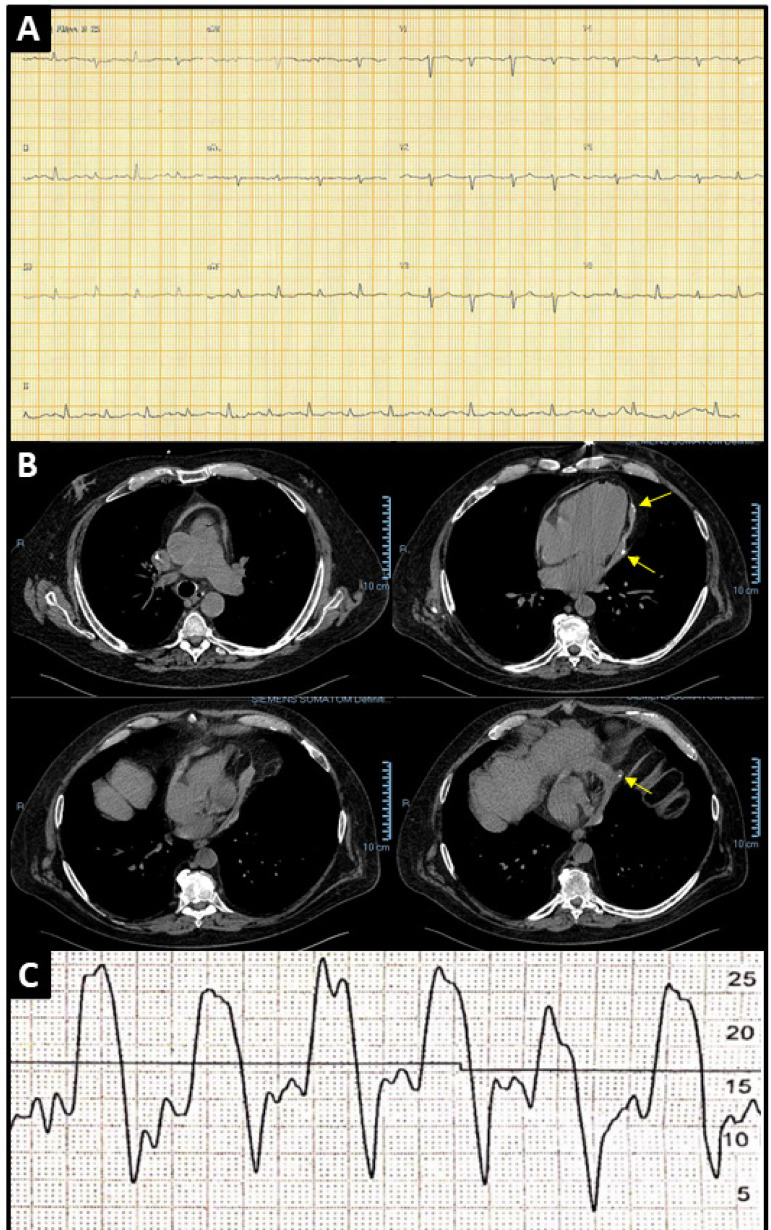
A) Electrocardiogram showing diffuse low voltage of QRS waves without evidence of ischemia or arrhythmia. B) Chest computed tomography scan showing pericardial thickening and foci of calcification (yellow arrows). C) Cardiac catheterization confirming a diagnosis of constrictive pericarditis and showing equalization of atrial and ventricular diastolic pressures and the square root sign.

Physical examination upon admission showed the patient was in poor general condition, pale and cyanotic. Lung auscultation was normal. The patient had a respiratory rate of 16 bpm and an oxygen saturation of 88% on ambient air. Cardiac auscultation revealed hypophonetic sounds, no murmurs, heart rate of 88 bpm, blood pressure of 50/30 mmHg with a prolonged capillary refill time and elevated jugular venous pressure. His abdomen was normal, and his legs had no sign of edema. The patient showed signs of confusion and sluggishness during the neurological examination. His electrocardiogram showed a sinus rhythm, diffuse low QRS-wave voltage, and altered ventricular repolarization (Figure 1A).

The patient was anuric and went into shock. Resuscitation was performed with 1,000 mL of crystalloid fluid, but his blood pressure did not recover to normal levels. Samples were cultured and the patient was started on vancomycin and ceftazidime until the etiology of shock was clarified. He was referred to the intensive care unit (ICU), where he was prescribed noradrenaline 0.2 mcg/kg/min. His clinical and neurological condition improved, despite the presence of pulsus paradoxus.

His workup results were as follows: hemoglobin: 9.8 g/dL; hematocrit: 29.5%; leucocytes: 5,230/mm^3^; platelets: 98,000/mm^3^; C-reactive protein: 0.61 mg/dL; BUN: 66.82 mg/dL; creatinine: 7.94 mg/dL; Na: 134 mEq/L; K: 6.2 mEq/L; ionized calcium: 1.17 mmol/L; P: 7.8 mg/dL; ALT: 14 U/L; AST: 22 U/L; alkaline phosphatase: 81 U/L; total bilirubin: 0.51 mg/dL; albumin: 3.9 g/dL; INR: 1.3; and aPTT: 1.29. Myocardial ischemia testing and posterior blood cultures were negative.

A transthoracic echocardiogram showed mild left atrial enlargement. His two ventricles had preserved systolic function, with an ejection fraction of 67% despite abnormal septal motion. The patient had a thickened pericardium without signs of effusion, along with hyperechogenicity areas. His inferior vena cava (IVC) was dilated and had no signs of inspiratory collapse. The association between mitral annulus tissue Doppler findings and IVC plethora suggested constrictive pericarditis. Chest computed tomography scans showed a thickened pericardium with gross calcification areas (Figure 1B). Cardiac catheterization showed equalization of diastolic pressures in all heart chambers and exhibited the square root sign (Figure 1C), indicative of constrictive pericarditis. An echocardiogram taken ten months prior to admission showed no evidence of pericardial abnormalities.

**Figure 2:**
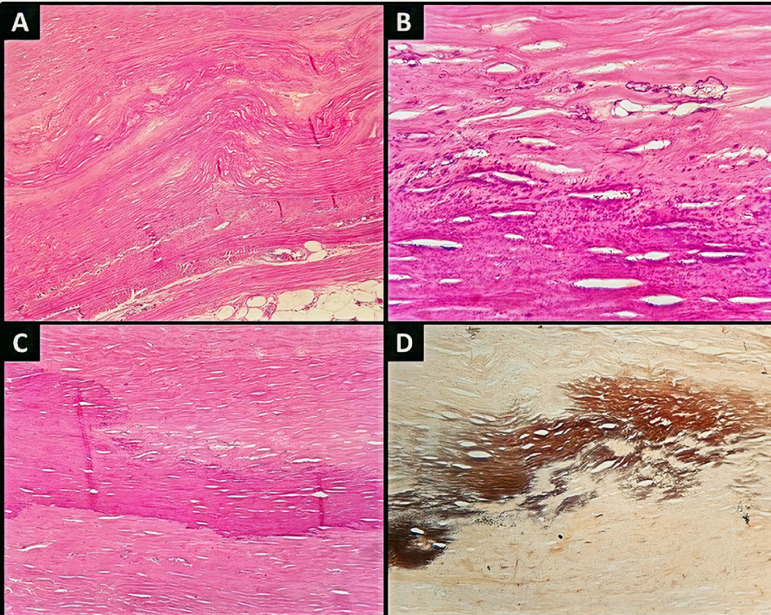
Photomicrographs showing: A) Thickened pericardium with fibrosis (hematoxylin and eosin, 50 x) associated with foci of dystrophic calcification (B and C, hematoxylin and eosin, 400x and 200x respectively). D) Areas of calcification are better seen with the Von Kossa stain (200x).

The patient underwent a pericardiectomy based on a diagnosis of constrictive pericarditis and cardiogenic shock. Pericardial fluid cultures were negative for fungal and bacterial infection, and the level of adenosine deaminase (ADA) was 23 U/L (normal range: <40U/L). Pericardial fluid culture and polymerase chain reaction (PCR) testing were negative for *Mycobacterium tuberculosis*. Histological analysis of the surgical specimen showed marked fibrosis, areas of dystrophic calcification, and mild foci of mononuclear inflammation (Figure 2), without signs of granuloma or infection. An investigation for systemic neoplasia and autoimmune diseases, and a tuberculin skin test came back negative.

After ruling out additional secondary causes, the combination of constrictive pericarditis and poor compliance to hemodialysis led to the diagnosis of Dialysis-related constrictive pericarditis (DRCP) The patient was prescribed a more intense hemodialysis protocol and was discharged nine days later. Since then, he has rigorously attended hemodialysis sessions and complied with his prescribed diet and fluid intake requirements.

## DISCUSSION

Pericardial disease is a potential consequence of fluid accumulation between parietal and visceral pericardial leaflets leading to cardiac tamponade, a condition in which the pericardial leaflets become stiffened by fibrosis and calcification, thereby producing constrictive events^4^^,^ 10. In most cases, constrictive pericarditis is the consequence of acute and chronic processes including surgery, exposure to radiation, pericardial bleeding, infection, autoimmune disease, use of medication, and uremia, to name a few^10^^-^ 12. Estimates point out that less than 10% of the cases of constrictive pericarditis are related to CKD and uremia^12^.

The pathophysiology of pericarditis associated with CKD includes uremia, a condition conducive to chronic inflammation status and serositis, hypervolemia, acid-base disorders, fluid and electrolyte disorders, calcium metabolism disorders, phosphorus metabolism disorders, and a baseline hypercatabolic state^4^^,^ 7 ^,^ 13.

Pericarditis cases related to CKD and uremia are divided into two subgroups: uremic pericarditis, when the condition develops before starting dialysis or up to eight weeks into dialysis and Dialysis-related constrictive pericarditis, a condition which develops from eight weeks of dialysis. The rationale behind the elected cut-off is that eight weeks should be enough for uremia and hypervolemia (two common conditions at the start of dialysis) to be resolved^4^^,^ 7 ^,^ 13.^( )^

Constrictive pericarditis usually manifests with signs of right heart failure including elevated jugular venous pressure, ascites, leg edema, tender hepatomegaly, hepatojugular reflux, pericardial knock, Kussmaul’s sign, and pulsus paradoxus. Signs of decreased systemic blood flow such as fatigue, lipothymia, prolonged capillary refill time, and arterial hypotension may be observed in more severe cases^10^^,^ 13.

In addition to the signs observed during physical examination, imaging findings further strengthened the case for constrictive pericarditis. Chest X-ray images showed pericardial calcifications, although this finding is not required for diagnosis of the condition. Electrocardiography revealed diffuse low QRS-wave voltage and non-specific alterations in T waves and ST segments. The patient’s echocardiogram showed a thickened pericardium (potentially associated with calcification), a dilated IVC without inspiratory collapse, a sudden stop in diastolic ventricular filling, and abnormal interventricular septal motion. Chest computed tomography and magnetic resonance imaging scans may show pericardial thickening, calcification, and pericardial fibrosis. A diagnosis of constrictive pericarditis may be based on typical echocardiography findings. Inconclusive echocardiograms may be supplemented with cardiac catheterization and right heart ventriculography to identify equalization of diastolic pressures in all heart chambers and demonstrate the square root sign, a pathognomonic finding of constrictive pericarditis^4^^,^ 10 ^-^ 12.

Individuals suspected of constrictive pericarditis must undergo careful examination and thorough investigation for autoimmune diseases, tumor, trauma, exposure to radiation, and infection^4^^,^ 7 ^,^ 10 ^,^ 14. Screening for *Mycobacterium tuberculosis* is imperative in Brazil and other countries in which tuberculosis is an endemic disease^14^^,^ 15. In the case reported herein, PCR testing and culture for *Mycobacterium tuberculosis* performed in pericardial fluid and surgical specimens came back negative.

Cardiology guidelines establish that the progression of dialysis-induced constrictive pericarditis is prevented with an intensification of dialysis. Therapy with non-steroid anti-inflammatory drugs may be considered for unresponsive patients. Individuals presenting signs of severe clinical deterioration such as hypotension and poor peripheral perfusion may be prescribed a pericardiectomy procedure^14^^,^ 15. In the case reported herein, the withdrawal of vasoactive drugs was only possible after surgery, thus confirming the severity of the disease and the need for an invasive procedure.

Cases of DRCP have been described to affect individuals on hemodialysis^7^^,^ 9 ^,^ 16 ^-^ 18 and peritoneal dialysis^13^^,^ 19 ^,^ 20. Reported cases generally date back to times when filters were not as efficient at removing solutes and patients remained uremic in spite of therapy^17^^,^ 18. As the efficiency of filters improved over the years and convective clearance was introduced, the number of cases of dialysis-induced constrictive pericarditis decreased dramatically^4^^,^ 7 ^,^ 13. Although a high-efficiency/high-flux filter was used and an appropriate Kt/V was defined in the case described in this paper, the patient often missed hemodialysis sessions and remained exposed to a uremic environment which was ultimately conducive to developing constrictive pericarditis. The solution involved educating the patient about the need to comply with therapy and adjusting his prescription to intermittent hemodiafiltration.

In conclusion, DRCP is an infrequent cause of hypotension and cardiogenic shock which may affect patients on hemodialysis and peritoneal dialysis. The condition is generally related to inadequate dialysis and may present progressive symptoms. Individuals on dialysis with hypotension of unknown cause should undergo echocardiographic examination.
